# Left ventricular morphology and function in rheumatoid arthritis patients without cardiac symptoms, using a cardiac magnetic resonance imaging

**DOI:** 10.1186/1532-429X-17-S1-P378

**Published:** 2015-02-03

**Authors:** Yasuyuki Kobayashi, Hitomi Kobayashi, Masaharu Hirano, Kihei Yoneyama, Yasuo Nakajima

**Affiliations:** 1 St.Marianna University School of Medicine, Kawasaki, Japan; 2 Itabashi Chuo Medical Center, Tokyo, Japan; 3 Johns Hopkins University School of Medicine, Baltimore, MD, USA; 4 Tokyo Medical School, Tokyo, Japan

## Background

Patients with rheumatoid arthritis (RA) experience an excess risk of congestive heart failure (CHF), chronic inflammation plays a pivotal role in this increased risk. Despite the growing body of research on cardiovascular (CV) disease in RA, the key pathways underlying the development of abnormalities in myocardial structure and function associated with CHF remain obscure. Cardiac magnetic resonance imaging (CMR) has been used to identify early functional and structural changes in the left ventricle (LV) before development of clinically overt CHF. We evaluated LV function and structure using a CMR in RA patients (pts) without cardiac symptoms, and determined the impact of non-biologic DMARDs and biologic DMARDs.

## Methods

Consecutive RA pts and healthy control were enrolled. RA pts received biologic DMARDs (bDMARDs) or non-biologic DMARDs (nbDMARDs). All subjects underwent evaluation of LV function and structure using non-contrast CMR. LV function was based on LV ejection fraction (EF), end-systolic volume (ESV), end-diastolic volume (EDV), stroke volume (SV), and cardiac output (CO). LV hypertrophy was measured by absolute LV mass (LVM) and LV mass index (LVMI) determined by LVM/body surface area. Subjects were classified into four categories based on LVMI and LVM/EDV of control subjects, with the mean + 2 SD of each measure defined as elevated LVMI and LVM/EDV.

## Results

We compared 90 female RA pts (mean age, 55.9±7.1 years) with a matched 20-patient control group (mean age, 52.7±4.6 years). 46 RA pts received nbDMARDs [43, methotrexate (MTX); 3, other drugs)] and 44 RA pts received bDMARDs [(18, infliximab; 26, tocilizumab) plus MTX]. The Simplified Disease Activity Index (SDAI) was significantly higher in the nbDMARDs group than in the bDMARDs group (*p*=0.002). Compared to the control group, the nbDMARDs group showed significantly higher LVMI and lower EF (*p*<0.001, *p*=0.003, respectively). There were no significant differences in LVMI and EF between the control and the bDMARDs groups. LV structure was classified as (1) concentric remodeling (LVMI<66.9 and LVM/EDV>1.02 (2) concentric hypertrophy (LVMI>66.9 and LVM/EDV>1.02 (3) eccentric hypertrophy (LVMI>66.9 and LVM/EDV <1.02 and (4) normal geometry (LVMI<66.9 and LVM/EDV<1.02. Among those with abnormal LV geometry, 32% of RA patients in the nbDMARDs group showed eccentric hypertrophy. 98% of RA patients in the bDMARDs group showed normal geometry. LVMI and EF were significantly associated with SDAI (r=0.567, *p*<0.001; r=0.412, *p*=0.02, respectively). Mass/EDV tended to be associated with SDAI (*p*=0.07). Adjustment for ESR did not modify the association of SDAI with EF and LVMI (*p*=0.017, *p*=0.005, respectively).

## Conclusions

Biologics treatment may reduce progression of subclinical LV dysfunction and normalize LV structure in association with the reduction in disease activity. It can be presumed that active RA may be an important contributor to the development of myocardial abnormalities.

## Funding

None.

